# Fleet behavior is responsive to a large-scale environmental disturbance: Hypoxia effects on the spatial dynamics of the northern Gulf of Mexico shrimp fishery

**DOI:** 10.1371/journal.pone.0183032

**Published:** 2017-08-24

**Authors:** Kevin M. Purcell, J. Kevin Craig, James M. Nance, Martin D. Smith, Lori S. Bennear

**Affiliations:** 1 National Marine Fisheries Service, Southeast Fisheries Science Center, Beaufort Laboratory, Beaufort, NC, United States of America; 2 National Marine Fisheries Service, Southeast Fisheries Science Center, Galveston Laboratory, Galveston, TX, United States of America; 3 Nicholas School of the Environment, Duke University, Durham, NC, United States of America; Louisiana State University, UNITED STATES

## Abstract

The northwestern Gulf of Mexico shelf experiences one of the largest seasonal hypoxic zones in the western hemisphere. Hypoxia (dissolved oxygen, DO ≤ 2.0 mg·L^-1^) is most severe from May to August during the height of the Gulf shrimp fishery, but its effects on the fishery are not well known. Prior studies indicate that hypoxia alters the spatial dynamics of shrimp and other species through habitat loss and aggregation in nearby oxygenated refuge habitats. We hypothesized that hypoxia-induced changes in the distribution of shrimp also alter the spatial dynamics of the Gulf shrimp fleet. We integrated data on the geographic distribution of shrimp tows and bottom DO to evaluate the effects of hypoxia on spatial patterns in shrimping effort. Our analyses indicate that shrimping effort declines in low DO waters on both the Texas and Louisiana shelf, but that considerable effort still occurs in low DO waters off Louisiana, likely because riverine nutrients fuel both benthic production and low bottom DO in the same general regions. The response of the shrimp fleet to hypoxia on the Louisiana shelf was complex with shifts in effort inshore, offshore, westward, and eastward of the hypoxic zone, as well as to an oxygenated area between two hypoxia regimes associated with the Mississippi and the Atchafalaya River outflows. In contrast, effort on the Texas shelf mostly shifted offshore in response to low DO but also shifted inshore in some years. Spatial patterns in total shrimping effort were driven primarily by the number of shrimp tows, consistent with aggregation of the fleet outside of hypoxic waters, though tow duration also declined in low DO waters. Overall, our results demonstrate that hypoxia alters the spatial dynamics of the Gulf shrimp fishery with potential consequences for harvest interactions and the economic condition of the fishery.

## Introduction

Marine and coastal ecosystems are increasingly exposed to environmental hypoxia (dissolved oxygen (DO) ≤ 2 mg·L^-1^), which has been documented in over 400 marine systems globally and affects > 240,000 km^2^ of coastal habitat [1]. Environmental hypoxia is a product of the excess consumption of oxygen through respiration and chemical processes relative to the rate of *in situ* oxygen production and inputs from other sources (e.g., atmospheric deposition). Hypoxia is especially common in shallow coastal systems with high nutrient inputs, long water residence times, and strong physical stratification [2]. Currently, the northwestern Gulf of Mexico experiences the largest seasonal hypoxic zone in the western hemisphere [3]. The areal extent of hypoxia in the Gulf has been estimated annually since 1985 and has exceeded 20,000 km^2^ in recent years [3, 4]. Hindcasting models and sediment paleo-indicators indicate low oxygen occurred in the Gulf beginning in the early 1900s but has become increasingly severe since the 1960s and 1970s [5–7]. Gulf hypoxia is driven by the combined effects of nutrient inputs, vertical salinity stratification associated with the outflows of the Mississippi-Atchafalaya River system, and regional and local oceanographic conditions [3, 8–11].

**Fig 1:**
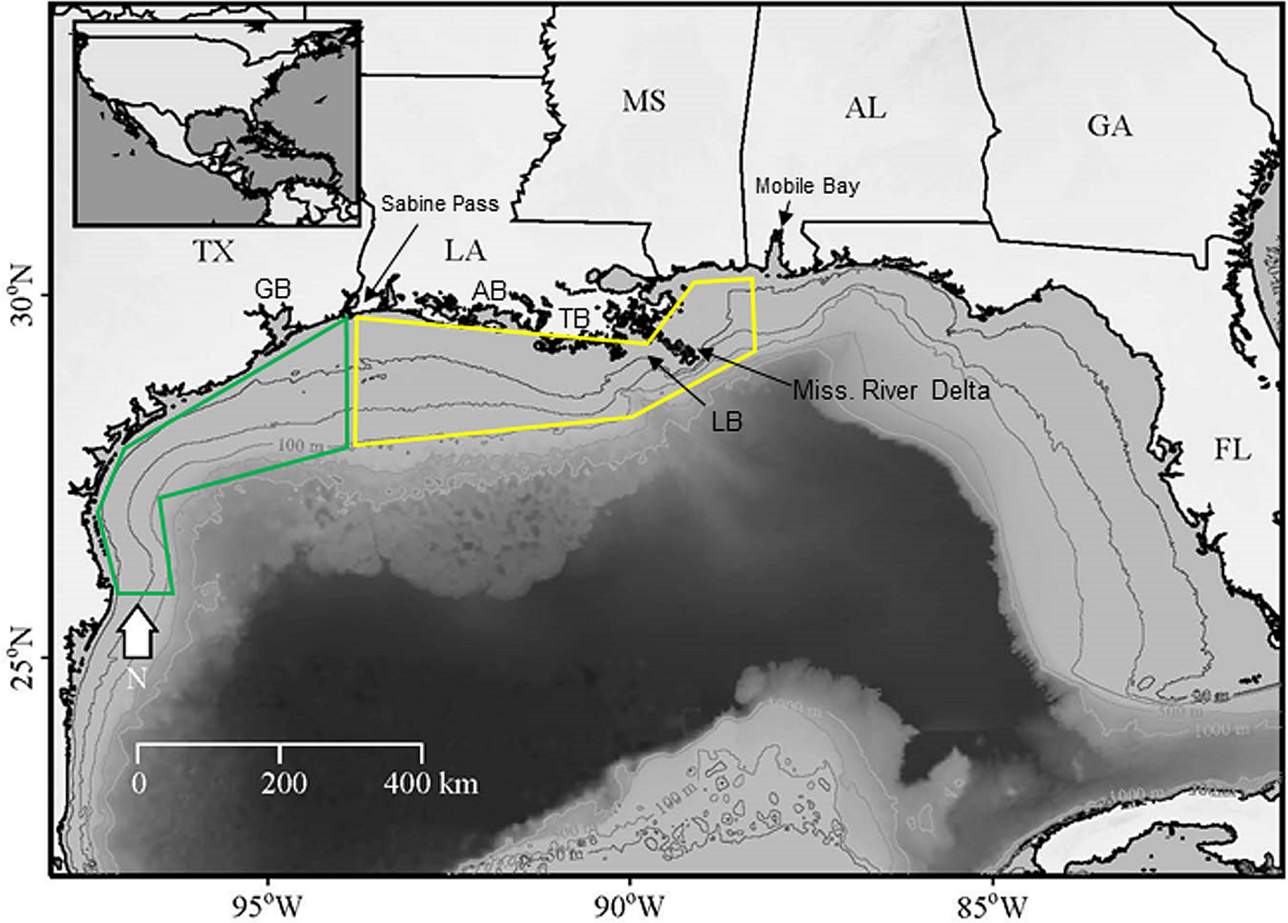
Study site map. Map of the northern Gulf of Mexico. Geographical reference points include Galveston Bay (GB), Atchafalaya Bay (AB), Terrebonne Bay (TB), the Louisiana Bight (LB) and the six bordering states. Mobile Bay and Sabine Pass mark the eastern and western extent of the “Fertile Fisheries Crescent” [12]. The Texas shelf is outlined in green and the Louisiana-Mississippi shelf is outlined in yellow.

The region of the northwestern Gulf associated with the Mississippi-Atchafalaya river plume has been referred to as the "Fertile Fishery Crescent" and supports some of the most productive fishing grounds in U.S. coastal waters (Fig 1) [12, 13]. The Gulf shrimp fishery is a bottom trawl fishery prosecuted in estuarine and coastal shelf waters from southern Florida to the Texas-Mexico border. The majority of shrimping effort occurs in the northwestern Gulf (Alabama to Texas) and predominantly targets two penaeid shrimp species: Brown Shrimp (*Farfantepenaeus aztecus*) and White Shrimp (*Litopenaeus setiferus*). The fishery began in the early 1900s as an inshore artisanal fleet and has grown to a large commercial fleet in excess of 20,000 vessels [14]. Historically, Gulf shrimp landings provided about 73% of the total shrimp landings in the US and the fishery was valued at > $500M USD annually, making it one of the most valuable US fisheries [15]. As recently as 2000, Brown Shrimp generated the highest revenues of any U.S. single-species fishery (http://www.st.nmfs.noaa.gov/st1/commercial/landings/annual_landings.html).

A primary response of mobile species exposed to hypoxia is to move to alternative habitats with higher DO levels [16–21]. While fish kills attributed to low DO have been reported in estuarine ecosystems in the Gulf [22], there is less evidence for direct mortality of mobile species in open shelf waters. Demersal fishes and shrimp in this region are relatively tolerant of low DO, with avoidance thresholds ranging from 1.06 to 1.99 mg·L^-1^ across ten of the most common species [19]. Despite the lack of strong geographic barriers to movement, species avoiding hypoxia aggregate at high densities within relatively short distances (e.g., ~ 5 km) from the edge of the Gulf hypoxic zone. This limited movement response to hypoxia may be due to locally enhanced benthic food resources that are fueled by the same riverine nutrients that drive the development of hypoxia [23]. Other studies in the Gulf have documented a general westward shift for some highly benthic species (e.g., flatfishes) to regions of the shelf with higher DO concentrations [18] as well as vertical displacement to the upper water column [24, 25].

Increased susceptibility to the Gulf shrimp trawl fishery is one potential indirect effect of hypoxia-induced habitat loss and aggregation of harvested species [19, 20, 26, 27]. If shrimpers are able to locate high-density aggregations of shrimp then catchability, or the harvest efficiency of a given unit of fishing effort, could increase [28]. For example, the Gulf menhaden purse seine fleet shifts inshore and to the west when hypoxia is severe [29]. Failure to incorporate the potential effects of these shifts in distribution on catchability into assessment models may lead to biased management advice [30]. A similar mechanism has been demonstrated for large pelagic species (i.e., tunas and marlins) that experience habitat compression and increased susceptibility to surface longline gear due to the vertical shoaling of deep water oxygen minimum zones [31, 32]. Bio-economic simulations of the Gulf shrimp trawl fishery suggest that hypoxia can lead to both short-term increases or decreases in catch, depending on the relative magnitude of hypoxia effects on components of shrimp production (e.g., growth, mortality) and the behavior of the fishery (e.g., catchability) [33]. The effects of hypoxia on size-based shrimp prices are consistent with increased catches of smaller shrimp during summer hypoxia and fewer shrimp escaping to larger size classes later in the year [34]. Collectively, these studies suggest complex and often ambiguous relationships between hypoxia, the performance of fisheries, and the models used for their management. To date, however, there have been no empirical studies on the fine-scale spatial dynamics of the Gulf shrimp fleet in response to hypoxia or other factors. A better understanding of the behavior of fishermen and the spatial response of the shrimp fleet to hypoxia is needed in order to evaluate potential consequences for the management and economic condition of the fishery.

In this study, we tested the hypothesis that large-scale hypoxia alters the spatial dynamics of the shrimp fleet on the northwestern Gulf of Mexico continental shelf. We integrated high-resolution data on the spatial distribution of individual shrimp tows with measurements of DO and other environmental variables from shipboard hydrographic surveys, and then used geospatial regression models to quantify the effects of hypoxia on the spatial distribution of shrimping effort in the northwestern Gulf. We developed separate models for the Louisiana shelf, a region of seasonally severe hypoxia, and the Texas shelf (reference site), a region with limited to moderate hypoxia. We also evaluated the effects of two underlying drivers of shrimping effort, tow density and the duration of individual shrimp tows. We interpret our results in terms of how environmentally-induced changes in the spatial distribution of harvested species can influence the spatial dynamics of fishing fleets with potential implications for harvest interactions.

## Methods

### Shrimping effort

**Fig 2:**
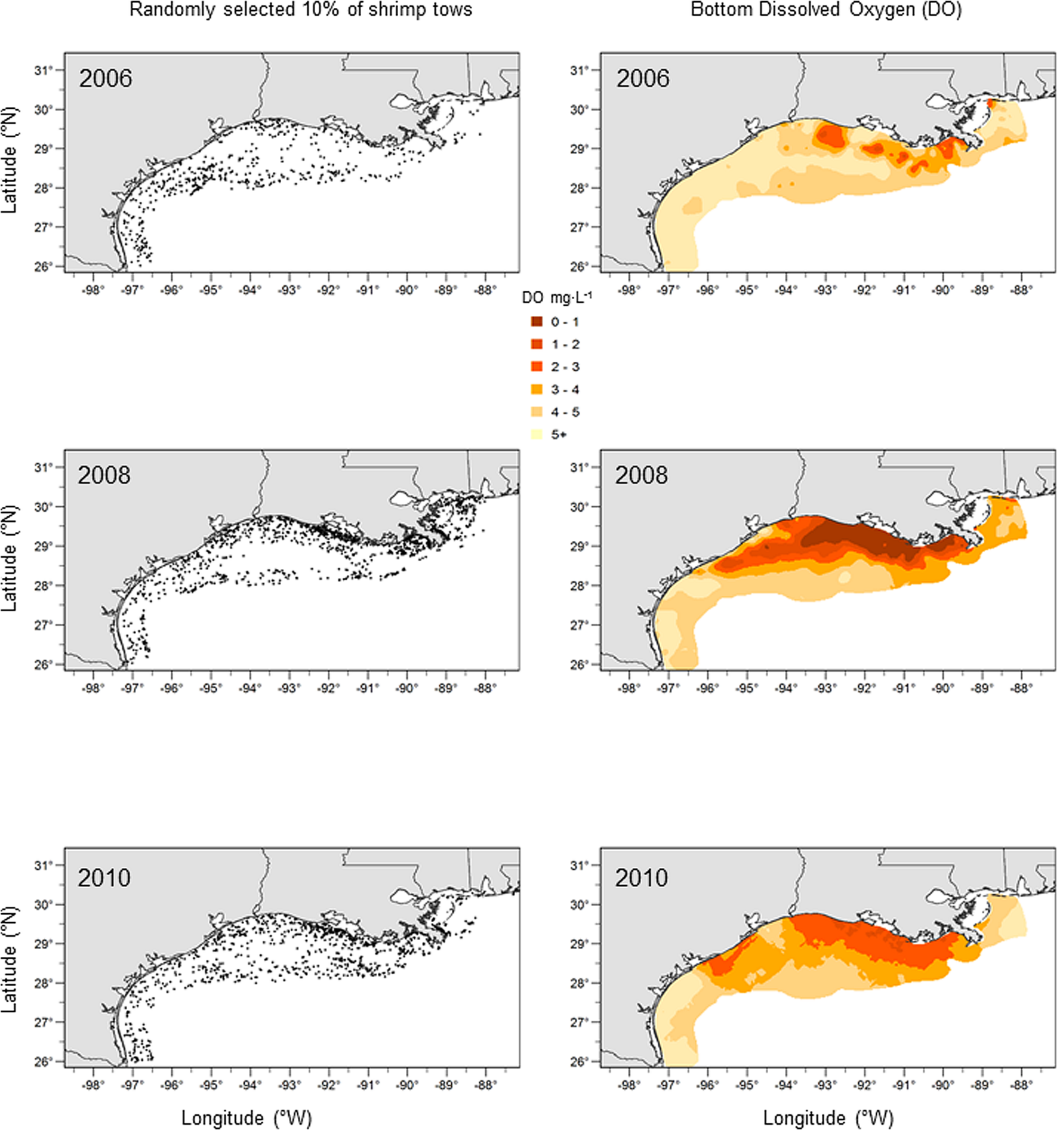
Shrimping effort and hypoxia. Maps showing the spatial distribution of a random sample (10%) of individual shrimp tows (left panels) and the spatial distribution of bottom dissolved oxygen (right panels) for three example years.

An electronic logbook (ELB) program was initiated in the northern Gulf of Mexico in 1999 to quantify spatial and temporal patterns in shrimping effort in the Gulf shrimp bottom trawl fishery [35]. A random sample of federally permitted shrimp vessels extending over nearly the entire geographic range of the fishery was instrumented with an ELB that recorded time and geographic location at a ten-minute sampling interval (Fig 2). Individual shrimp tows were identified based on changes in vessel speed, which are typically 4–5 times slower when the shrimp nets are deployed (2–3 knots) than during other activities (i.e., steaming; 8–10 knots). The starting location (latitude, longitude) of each tow was used to assign a geographic position and tow duration was used as a measure of shrimping effort. Comparison of data from ELBs with data from self-reported paper logbooks and from observer records indicate this methodology reflects spatial patterns in shrimping effort on the shelf [36]. The initial years of the ELB program instrumented a small number of vessels and were considered pilot studies. We used ELB data collected over 7 years (2004–2010) and during the summer period (June to August) (Fig 2). This dataset consisted of 124,222 individual tow records identified by date, tow duration (hours), and a starting latitude and longitude (Table 1). Vessels instrumented with an ELB represented on average 22% of federally permitted vessels in the Gulf shrimp fleet over this time frame.

**Table 1:** Electronic logbook (ELB) data used in the analysis.

Year	No. Vessels	% of Fleet	No. of Tows	Total Annual Effort (Vessel-Days)	Area Hypoxia (km^2^)
2004	67	2.39	2,037	1,46,623	15,700
2005	168	6.61	6,577	1,876,978	10,200
2006	378	17.0	12,067	1,715,535	15,600
2007	529	24.5	18,971	1,563,672	20,600
2008	666	35.1	31,592	1,192,488	22,300
2009	645	34.1	35,639	1,415,538	7,100
2010	573	32.3	17,339	1,230,499	15,600

Area Hypoxia (DO ≤ 2.0 mg l^-1^) taken from [50].

### Environmental data

Bathymetric and hydrographic conditions on the northwestern Gulf of Mexico shelf during summer (June to August) were characterized using data from the Southeast Area Monitoring and Assessment Program (SEAMAP) [37]. SEAMAP is a collection of fishery-independent surveys that have measured hydrographic conditions in association with biological sampling on the northwestern Gulf of Mexico shelf (-97.3° to -88.0° west longitude, 3–110 m depth) since 1987 (http://www.gsmfc.org/seamap.php). Bottom environmental conditions (e.g., temperature, salinity, DO, depth) were measured at approximately 300 point locations each summer using a conductivity, temperature, depth (CTD) profiler. Prior studies have used these data to quantify spatial patterns in bottom DO and environmental associations for several demersal species [23, 25, 27, 38, 39]. Additional details regarding the SEAMAP surveys and sampling procedures can be found in Eldridge [37] and the annual SEAMAP atlases [40].

Interpolated maps of summer (June-August) bottom DO (mg·L^-1^) were generated for each year of ELB data over the northwestern Gulf shelf (Fig 2). Following earlier studies [27], bottom water DO was interpolated separately for each year (2004–2010) using universal kriging with a quadratic drift component and a variable search radius with a sample count of at least 12 nearest neighbors. Water column depth was interpolated using inverse distance weighting of all SEAMAP records for surveys from 2004–2010. Bottom temperature and salinity were considered in preliminary analyses, but were removed due to high correlations with water depth. Because individual shrimp tows can extend over four hours and vary in direction [41], interpolations of bottom DO were smoothed prior to matching with shrimp tow locations. A square 3 x 3 neighborhood matrix of 5 km^2^ cells was defined with the grid cell of interest located in the center. For each cell in the interpolated surface, the predicted DO values were averaged across the surrounding spatial neighborhood. Smoothing had little effect on the interpolated surface because interpolated DO values varied little at this spatial scale (5–15 km) and the area of smoothing was small relative to the area of the interpolation (~ 133,000 km^2^). All interpolation and smoothing was conducted in ArcMap v. 10.4 (ESRI, Redlands, CA).

### Combining datasets

Individual shrimp tows from the ELB data were assigned bottom DO and depth values by sampling the smoothed surfaces of hydrographic and bathymetric data based on the geographic location of each tow. Hydrographic data collected from early June to late August in coastal waters from the Texas-Mexico border to the Mississippi-Alabama border were stratified by depth and by a series of 21 statistical zones delineated by 1° longitude in the east to west direction and 1° latitude in the north to south direction [42]. To match shrimping effort with the most synoptic environmental data, ELB tows were filtered to retain only those tows that were conducted within the same statistical zone and within the approximate two-week period (i.e., 10–14 days) that the environmental data was collected.

### Additional explanatory variables

Two economic variables that influence fishing behavior, shrimp market price per pound (*pPND*) and average weekly diesel fuel price (*pGAL*), were also included in the analysis [43]. Shrimp prices are maintained by the National Marine Fisheries Service (NMFS) Galveston laboratory as part of the shrimp fishery monitoring program [41]. The diesel fuel time series (Ultra-low sulfur CARB diesel spot price) was taken from the U. S. Energy Information Administration (http://www.eia.gov). This continuous fuel time series consists of daily spot prices in US dollars for diesel fuel in Los Angeles, CA since 1996, and is highly correlated (r^2^ = 0.98) with a similar fuel price time series for the Gulf of Mexico region that did not cover the entire period of interest (2004–2010).

### Regression models

We used generalized additive models (GAMs) to investigate relationships between shrimping effort and environmental variables (DO, depth) using both spatially-varying and spatially-invariant model formulations [44–46]. All models were structured with a dependent variable representing a measure of shrimping effort, *X*_*d*,*y*,(*ρ*,*φ*)_ on Julian day (*d*) in year (*y*) for a specific grid cell with a centroid located at latitude (*ρ*) and longitude (*φ*):

$$X_{d,y,(\rho ,\varphi )}\;={a+\alpha }_{1}\;(y)+\alpha_{2}\;(pFuel)+\alpha_{3}\;(Effort)+g_{1}\;(DO)+g_{2}\;(Depth)+g_{3}\;(pShrimp)+g_{4}\;(d)+g_{5}\;(\rho ,\varphi )+e_{d,y,(\rho ,\varphi )}$$ (1)

**Fig 3:**
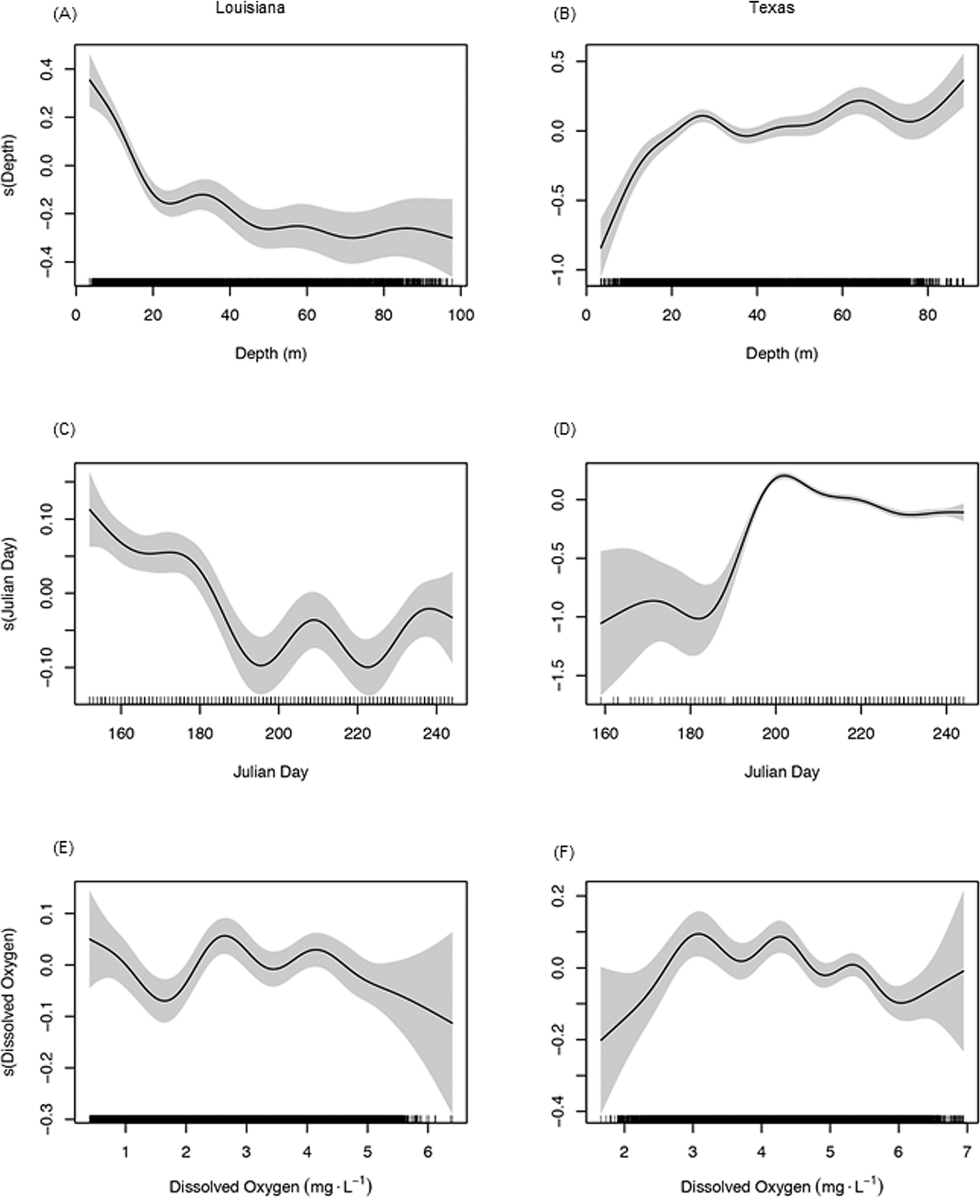
Effects of predictor variables on shrimping effort. Smooth plots from regression models showing the effect of (A,B) Depth (m), (C,D) Julian day, and (E,F) Bottom dissolved oxygen (DO, mg^-1^∙L) on total shrimping effort. Left column plots are for the Louisiana model and right column plots are for the Texas model. The y-axis shows the partial effect of each predictor variable on shrimping effort across the range of the predictor shown on the x-axis. The shaded area represents the 95% confidence intervals about the fitted curve. The rug plot on the x-axes represents individual data points.

The spatially-invariant model (Eq 1), included nonparametric smooth terms (*g*_*x*_) for dissolved oxygen (*DO*) in mg·L^-1^, depth (*Depth*) in meters, average weekly shrimp price per pound (*pShrimp*) in USD, day of the year (*d*), and the latitude (ρ) and longitude (φ) of each grid cell centroid. These factors were modeled as nonparametric smoothed terms in order to isolate the effect of DO from other factors that are potentially correlated (e.g., depth) or also influence shrimping effort (e.g., shrimp prices). The smoothed plots (e.g., Fig 3) represent the relationship between the response variable (e.g., shrimping effort) and each predictor variable (e.g., DO) after accounting for the effect of other terms in the model (e.g., depth, shrimp prices), and are analogous to model coefficients in traditional multiple regression. The response variable (y-axis) is on the transformed scale (via the link function) so that the cumulative effects of the predictors on the response at any given value can be calculated as a sum of the partial effects [46]. The amount of smoothing in these plots is based on the degrees of freedom (df) of the smoothing parameter, which is optimized as part of the fitting routine, so that peaks and valleys reflect the best estimate of the partial effect of each predictor on shrimping effort. Effects of each predictor are strongest in regions of the curve with relatively narrow confidence intervals, which typically reflect samples sizes (shown on the rugplot along the x-axis). All smoothed predictors used thin plate regression splines and were modeled as 1-dimensional smoothing functions except for location (*ρ*,*φ*), which was modeled with a 2-dimensional smoothing function [46]. The effects of year (*y*), average weekly fuel price (*pFuel*), and total annual shrimping effort (*Effort*) were modeled as parametric (i.e., not smoothed) terms (*α*_*x*_) and *e*_*d*,*y*,(*ρ*,*φ*)_ is a model specific error term. The nonparametric terms reflect factors that might also explain variation in shrimping effort, but were either not measured directly (e.g., actual fuel prices paid by individual shrimp vessels at particular times during the season are unknown) or were measured at a different temporal scale (e.g., annual shrimping effort) than the smoothed terms. The spatially-invariant model (Eq 1) assumes the effect of the predictor variables on shrimping effort are the same over the entire shrimping grounds.

The spatially-varying model formulation (Eq 2) is similar to the spatially-invariant model except that the *DO* effect is modeled as a 2-dimensional smoothing function that allowed the dissolved oxygen (*DO*) coefficient to vary smoothly as a function of space (*ρ*,*φ*):

$$X_{d,y,(\rho ,\varphi )}\;=a+\alpha_{1}\;(y)+\alpha_{2}\;(pFuel)+\alpha_{3}\;(Effort)+g_{1}\;(Depth)+g_{2}\;(pShrimp)+g_{3}\;(d)+g_{4}\;(\rho ,\varphi )+g_{5}\;(\rho ,\varphi )\;DO+e_{d,y,(\rho ,\varphi )}$$ (2)

Variable-coefficient models that map variation in the relationship between a response variable and a predictor variable across space are referred to as geospatial regression models and have recently been applied in a number of ecological studies [45, 47–49]. The spatially varying term (*g*_*5*_*(ρ*,*φ) DO*) is similar to an interaction term in traditional regression in which the DO effect is allowed to vary across space. The strength of the variable coefficient GAM approach is that specific areas of the shelf where shrimping effort increases or decreases with a unit decrease in DO can be identified.

A quasi-experimental framework was used where one model was developed for the area of the shelf that experiences recurrent bottom water hypoxia during summer (Louisiana and part of Mississippi coast; 94.0° – 88.0° west longitude; hereafter, Louisiana shelf), and a separate model was developed for a reference site. We considered the Texas shelf (97.3° - 94.0° west longitude) a reference site because it experiences limited hypoxia during summer. Spatially-varying and spatially-invariant models were developed separately for the two regions for the summer period (June to August) over the seven years when ELB data were available (2004–2010). Three response variables that reflect different aspects of fishing behavior were computed: total shrimping effort, average tow duration, and tow density. Each response variable was aggregated within a 10 x 10 minute spatial grid extending across the northwestern Gulf of Mexico shelf separately for each year (2004–2010). Total shrimping effort was the total number of hours towed by all vessels within a grid cell in each year. Average tow duration was computed across all tows within a grid cell for each year, and tow density was the total number of tows conducted within each grid cell for each year. Total shrimping effort and average tow duration were log (x+1) transformed and modeled with a Gaussian error distribution while tow density was modeled assuming a Poisson error distribution. Interpolated environmental variables (DO, Depth) were also averaged within each spatial grid cell separately for each year. The spatially-varying model formulation (Eq 2) was also run separately for the two years with the largest and the smallest spatial extent of hypoxia for each of the regions (i.e., Texas, Louisiana): In 2008, hypoxia extended over 22,300 km^2^ while in 2009 hypoxia extended over only 7,100 km^2^ [50]. The model formulation for each of these two years was identical to (Eq 2) except that no year effect was included. Generalized cross validation (GCV), an approach to limit model overfitting by minimizing a roughness penalty function [45], was used to automate selection of the smoothing parameter (lower GCV score indicates better fit) for all models. All regression models were developed in the R 2.14.2 statistical computing environment using the R package mgcv 1.7–13.

## Results

Spatially-varying and spatially-invariant model formulations were similar in terms of effect sizes and the statistical significance of the predictor variables (Table 2). All factors were statistically significant except for individual year effects in some models. One-dimensional smoothed terms were highly nonlinear, with degrees of freedom ranging from six to nine. The effect of bottom DO on total shrimping effort was significant and highly nonlinear for all models, even after accounting for correlated effects and other factors that influence the fishery. Spatially-varying models were marginally better for both the Louisiana and the Texas shelf based on generalized cross validation (GCV) scores.

**Table 2:** Spatially-invariant and spatially-varying regression model results for shrimping effort on the Louisiana and Texas continental shelf.

	Louisiana		Texas	
Term	Invariant	Varying	Invariant	Varying
Parametric Intercept (a)	**1.71 (0.12)**	**1.79 (0.12)**	**1.09 (0.10)**	**1.05 (0.11)**
2005	0.01 (0.08)	0.02 (0.08)	**0.19 (0.03)**	**0.15 (0.04)**
2006	**0.34 (0.06)**	**0.37 (0.06)**	**0.26 (0.04)**	**0.23 (0.04)**
2007	**0.53 (0.05)**	**0.54 (0.05)**	**0.20 (0.04)**	**0.19 (0.04)**
2008	**0.91 (0.08)**	**0.98 (0.08)**	**0.39 (0.09)**	**0.38 (0.09)**
2009	**0.81 (0.03)**	**0.82 (0.03)**	**0.65 (0.02)**	**0.64 (0.03)**
2010	**0.77 (0.03)**	**0.79 (0.03)**	**0.52 (0.03)**	**0.53 (0.03)**
pFuel	**-0.08 (0.04)**	**-0.11 (0.04)**	**-0.19 (0.05)**	**-0.19 (0.05)**
Effort	**2.0e**^**-7**^ **(8.4e**^**-8**^**)**	**2.2e**^**-7**^ **(8.5e**^**-8**^**)**	**2.1e-7 (2.1e**^**-8**^**)**	**2.0e**^**-7**^ **(2.6e**^**-8**^**)**
Smoothed s(DO)	**6.73 (7.55)**		**7.41 (7.89)**	
s(Depth)	**7.07 (7.74)**	**7.04 (7.73)**	**7.91 (7.96)**	**6.22 (7.22)**
s(pShrimp)	**6.29 (7.24)**	**6.26 (7.22)**	**6.81 (7.59)**	**6.99 (7.70)**
s(Julian Day)	**6.87 (7.62)**	**6.90 (7.64)**	**7.51 (7.87)**	**7.84 (7.98)**
s(Lat, Lon)	**26.9 (27.9)**	**27.7 (28.0)**	**27.1 (28.0)**	**24.4 (25.6)**
s(Lat, Lon):DO		**26.7 (29.2)**		**22.5 (25.8)**
GCV score	0.80	0.79	0.71	0.70

DO, dissolved oxygen; pShrimp, shrimp price; pFuel, fuel price; GCV generalized cross validation score (lower score indicates better model fit).

Model coefficients and standard errors in () are shown for parametric terms.

Estimated degrees of freedom (df) and reference degrees of freedom prior to smoothing penalty in () are shown for smoothed (s) terms.

The degrees of freedom reflect the amount of smoothing in the model (higher df leads to less smoothing) and is optimized during the fitting routine.

Model coefficients with *p* ≤ 0.05 are in bold.

Most shrimping effort on the Louisiana shelf occurred in shallow nearshore waters with effort declining sharply at depths > 20 m (Fig 3A). On the Texas shelf, little shrimping effort occurred in shallow, nearshore waters with effort increasing with increasing depth, particularly from shore to depths of 30 m (Fig 3B). Shrimping effort off Louisiana was highest during the early summer (prior to early July, < Julian day 184) and declined by late summer (Fig 3C). In contrast, effort was low during the early summer off Texas and increased sharply by mid-summer (mid-July ~julian day 196) (Fig 3D). These differences in the seasonal timing and depth distribution of the fishery reflect different management objectives for the two regions. Texas manages for an offshore fishery by imposing an early season federal closure (no harvest prior to ~July 15) so that harvest is restricted until later in the summer when shrimp are larger and typically occur in deeper waters [51]. The Louisiana fishery is not subject to a closure or catch limits and so harvests shrimp earlier in the season when they are smaller and emigrating from estuarine waters to the nearshore shelf.

The effect of DO on shrimping effort also differed between the two regions (Fig 3E and 3F). On the Louisiana shelf, the highest effort occurred at intermediate DO levels, but considerable effort still occurred in hypoxic waters (Fig 3E). In fact, while effort on the Louisiana shelf was lowest at 2 mg·L^-1^, effort increased with a further decrease in DO to < 1 mg·L^-1^, though uncertainty at these low DO levels was high. On the Texas shelf, where hypoxia is much less severe and spatially limited, shrimping effort declined sharply at DO < 3 mg·L^-1^ and the highest effort occurred at intermediate DO levels (3–4 mg·L^-1^) (Fig 3F). Overall, at levels above 2 mg·L^-1^ there was a general downward trend in shrimping effort with increasing DO for both the Louisiana and the Texas shelf.

**Fig 4:**
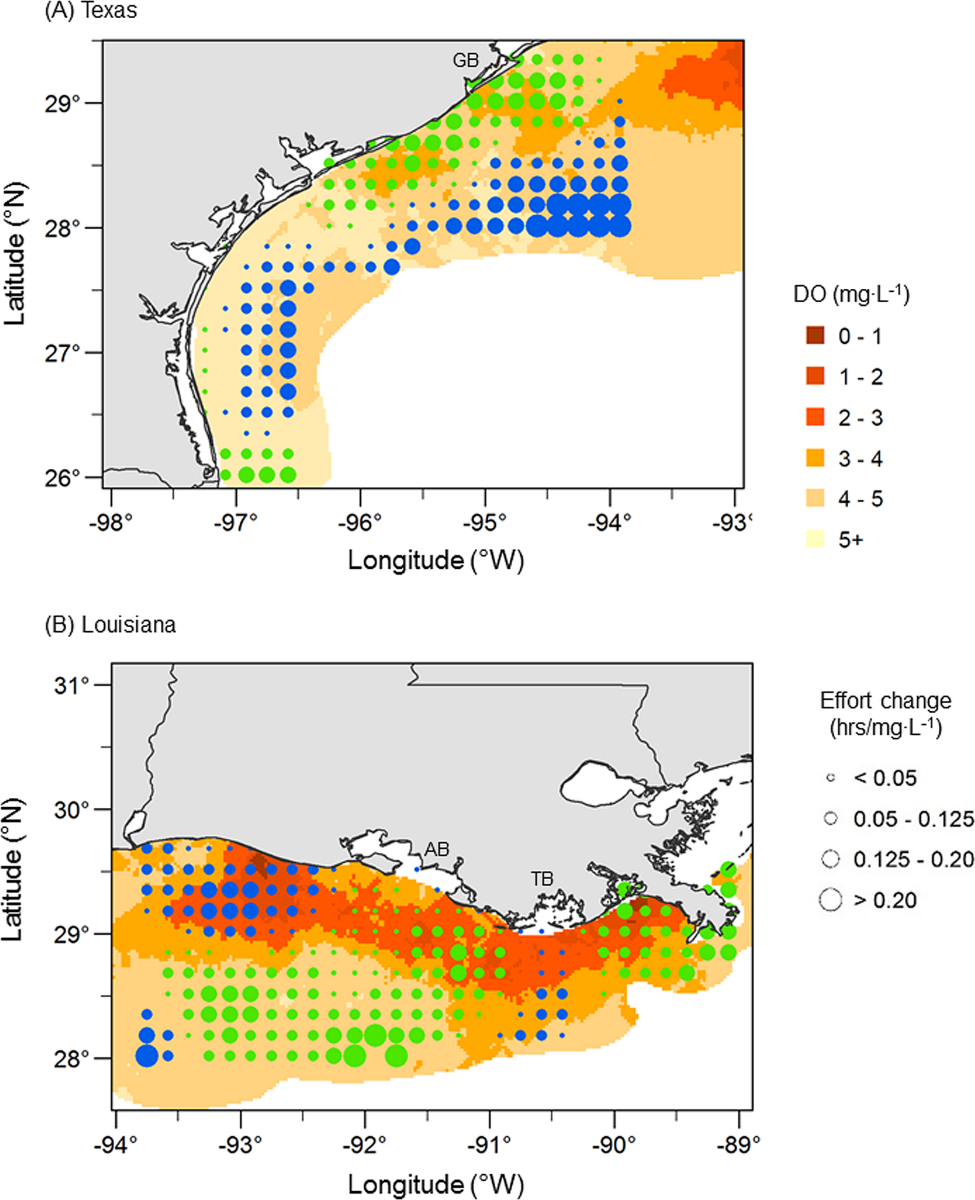
Fine-scale spatial effects of hypoxia on shrimping effort. Geospatial regression model estimates of the spatially varying effect of dissolved oxygen (DO) on shrimping effort for (A) the Texas shelf and (B) the Louisiana shelf during the period 2004–2010. Circles represent the local rate of change in shrimping effort with bottom DO, with circle size proportional to the magnitude of the positive (green, effort declines as DO declines) and negative (blue, effort increases as DO declines) effects. All other terms in the model were held constant at their mean values in order to isolate spatial patterns in the effect of DO on shrimping effort. The surface is an interpolation of average bottom DO conditions based on data pooled over the years 2004–2010 and may not reflect a particular year. Geographical reference points include Galveston Bay (GB), Atchafalaya Bay (AB), and Terrebonne Bay (TB).

The effect of DO on shrimping effort varied spatially on both the Texas and the Louisiana shelf (Fig 4). When DO declined, effort declined in shallow nearshore waters on the Texas shelf and increased in deeper offshore waters. This effect was consistent across nearly the entire Texas shelf, but was strongest on the north Texas shelf offshore of Galveston Bay near the Louisiana border (Fig 4A). In contrast, DO effects on the Louisiana shelf were more complex with multiple interspersed regions where shrimping effort was both positively and negatively related to bottom DO (Fig 4B). When DO declined on the Louisiana shelf, shrimping effort declined in the Louisiana Bight, a region of chronically severe hypoxia, as well in waters directly off Atchafalaya Bay. Shrimping effort increased in nearshore waters west of Atchafalaya Bay to the Texas border and in limited areas offshore as well as in waters directly off Terrebonne Bay. The latter is a region that sometimes experiences oxygenated bottom water surrounded by two distinct regions of hypoxia associated with the separate Mississippi and Atchafalaya River outflows [9, 50].

**Fig 5:**
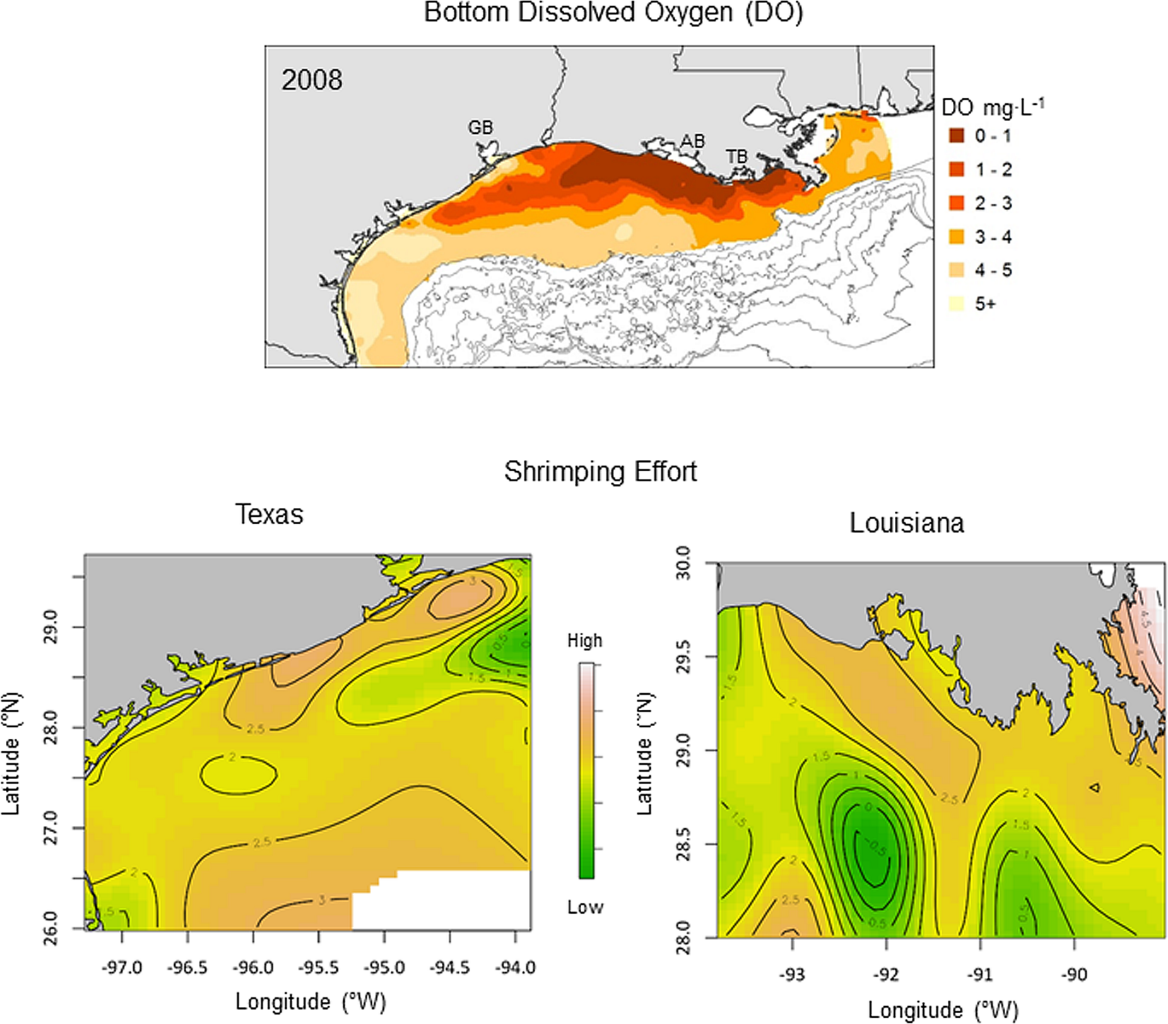
Spatial distribution of shrimping effort during severe hypoxia. Spatial distribution of bottom dissolved oxygen (DO, top panel) and shrimping effort on the Texas (bottom left panel) and Louisiana (bottom right panel) continental shelf in 2008. Map surfaces and contours represent estimated shrimping effort from the geospatial regression model. Geographical reference points include Galveston Bay (GB), Atchafalaya Bay (AB), and Terrebonne Bay (TB).

The spatial distribution of shrimping effort also differed regionally (i.e., Texas vs. Louisiana) between the years with the largest (2008: 22,300 km^2^) and smallest (2009: 7,100 km^2^) areal extent of hypoxia. During the severe hypoxia year (2008), hypoxia extended well into coastal waters off Texas (Fig 5). Shrimping effort on the Texas shelf was highest in nearshore waters along the north Texas coast inshore of the hypoxic zone. Effort on the Louisiana shelf was concentrated in nearshore shelf waters as well, with very low effort occurring in two distinct areas off Terrebonne and Atchafalaya Bays. Estimated effort contours were closely spaced in both regions, indicating that low DO caused relatively rapid changes in shrimping effort over small distances.

**Fig 6:**
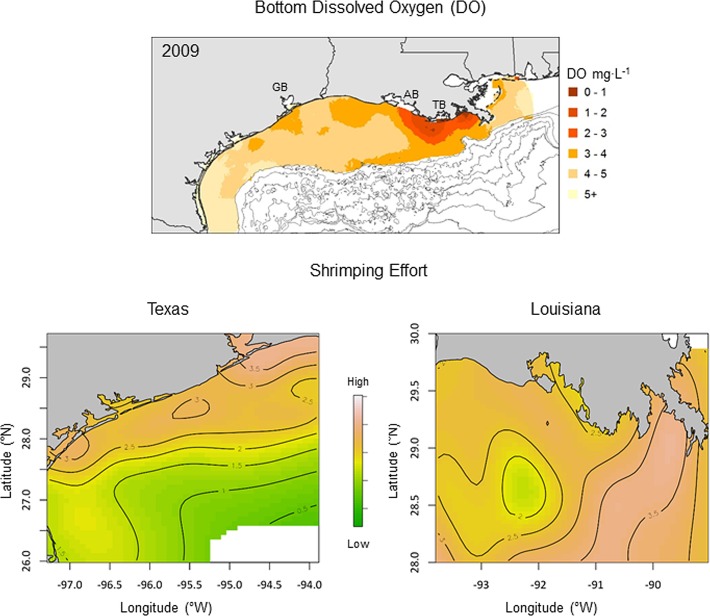
Spatial distribution of shrimping effort during moderate hypoxia. Spatial distribution of bottom dissolved oxygen (DO, top panel) and shrimping effort on the Texas (bottom left panel) and Louisiana (bottom right panel) continental shelf in 2009. Map surfaces and contours represent estimated shrimping effort from the geospatial regression model. Geographical reference points including Galveston Bay (GB), Atchafalaya Bay (AB), and Terrebonne Bay (TB).

In contrast, during the moderate hypoxia year (2009) low DO was limited to east of Atchafalaya Bay (Fig 6). The highest shrimping effort on the Louisiana shelf occurred in some of the same areas that experienced the lowest effort in the previous year (2008). Similar to the severe hypoxia year, shrimping effort was highest on the north Texas shelf in 2009, but was more evenly distributed along the coast. Contours of shrimping effort were more broadly spaced for both regions in 2009 compared to 2008, indicating less spatial structure and a more broadly distributed fishery in the moderate compared to the severe hypoxia year.

**Fig 7:**
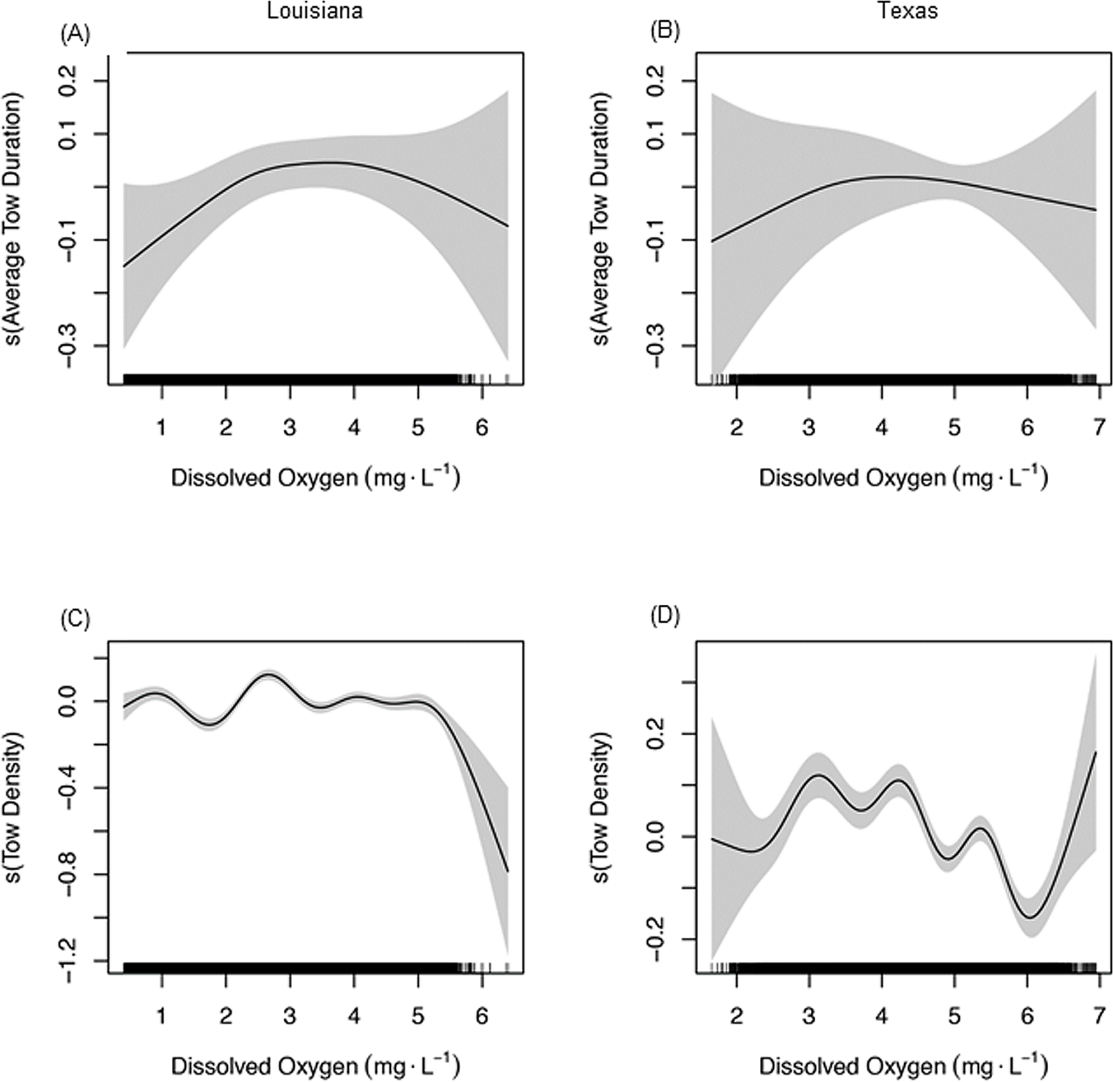
Effects of predictor variables on tow duration and tow density. Smooth plots from regression models showing the effect of bottom dissolved oxygen (mg^-1^∙L) on (A,B) average tow duration and (C,D) tow density for the Louisiana and Texas continental shelf. The y-axis shows the partial effect of each predictor variable on the response (tow duration or tow density) across the range of the predictor shown on the x-axis. The shaded area represents the 95% confidence intervals about the fitted curve. The rug plots on the x-axes represent individual data points.

Models for average tow duration and tow density showed significant or marginally significant effects for most model parameters (Table 3). Similar to total shrimping effort, one-dimensional smoothed parameters were highly nonlinear, with degrees of freedom ranging from four to nine. The average duration of a tow on the Louisiana shelf was stable across a broad range of DO levels (0.5–5 mg·L^-1^), with a decline below 2 mg·L^-1^ and a peak near 3–4 mg·L^-1^ (Fig 7A). A similar but weaker effect of DO on average tow duration was evident on the Texas shelf (Fig 7B). Models for tow density were more complex but showed similar patterns with DO to that observed for total shrimping effort (compare Fig 7C and 7D and Fig 3E and 3F). For example, on the Louisiana shelf average tow density was low at 2 mg·L^-1^ and increased at both lower (< 1 mg·L^-1^) and higher (3 mg·L^-1^) DO levels (Fig 7C), similar to the pattern observed for total shrimping effort (Fig 3E). On the Texas shelf, average tow density was high at intermediate DO levels (3–4 mg·L^-1^) and declined at both lower (< 3 mg·L^-1^) and higher (4–6 mg·L^-1^) DO levels (Fig 7D), similar to the pattern in total shrimping effort observed in Texas waters (Fig 3F). For both regions, tow density was highest near 3 mg·L^-1^ and showed a declining trend at higher DO levels, with the exception of an increase at DO > 6 mg·L^-1^ off Texas.

**Table 3:** Regression model results for average tow duration and tow density on the Louisiana and Texas continental shelf.

	Tow Duration		Tow Density	
Term	Louisiana	Texas	Louisiana	Texas
Parametric Intercept (a)	**3.62 (0.32)**	**2.99 (0.32)**	NA	NA
2005	**-0.56 (0.20)**	-0.13 (0.11)	**0.34 (0.09)**	**0.23 (0.04)**
2006	**0.63 (0.17)**	**0.60 (0.13)**	**0.51 (0.08)**	0.07 (0.05)
2007	**1.32 (0.13)**	**0.61 (0.11)**	**0.63 (0.08)**	0.02 (0.05)
2008	**1.99 (0.22)**	**1.61 (0.25)**	**1.23 (0.09)**	0.05 (0.10)
2009	**1.75 (0.09)**	**1.47 (0.08)**	**0.97 (0.07)**	**0.46 (0.05)**
2010	**1.74 (0.07)**	**1.60 (0.09)**	**1.07 (0.06)**	**0.24 (0.05)**
pFuel	-0.16 (0.11)	0.02 (0.15)	**-0.14 (0.02)**	**0.24 (0.03)**
Effort	**4.6e**^**-7**^ **(2.2e**^**-7**^**)**	**3.1e**^**-7**^ **(6.1e**^**-8**^**)**	**3.8e-7 (4.8e-8)**	**1.5e**^**-7**^ **(1.8e**^**-8**^**)**
Smoothed s(Depth)	**6.80 (7.60)**	**5.90 (6.96)**	**8.79 (8.98)**	**8.69 (8.97)**
s(pShrimp)	2.53 (3.22)	**5.47 (6.53)**	**8.12 (8.80)**	**8.23 (8.84)**
s(Julian Day)	**4.53 (5.56)**	**7.05 (7.60)**	**8.88 (8.99)**	**8.76 (8.96)**
s(Lat, Lon)	**27.3 (27.8)**	**27.7 (28.0)**	**28.9 (29.0)**	**29.9 (29.0)**
s(Lat,Lon):DO	**26.8 (29.1)**	**26.7 (29.2)**	**29.2 (29.9)**	**27.3 (29.5)**
GCV score	5.54	5.88	1.08	0.80

DO, dissolved oxygen; pShrimp, shrimp price; pFuel, fuel price; GCV generalized cross validation score (lower score indicates better model fit).

Model coefficients and standard errors in () are shown for parametric terms.

Estimated degrees of freedom (df) and reference degrees of freedom prior to smoothing penalty in () are shown for smoothed (s) terms.

The degrees of freedom reflect the amount of smoothing in the model (higher df leads to less smoothing) and is optimized during the fitting routine.

Model coefficients with *p* ≤ 0.05 are in bold.

## Discussion

Geospatial analysis of electronic logbook (ELB) data revealed that hypoxia alters the spatial dynamics of the northwestern Gulf of Mexico shrimp trawl fleet during summer, when both the shrimp fishery and hypoxia severity are at their seasonal peaks. Relationships between bottom DO and shrimping effort were highly significant and nonlinear even after accounting for other environmental (e.g., depth) and economic (e.g., fuel price) factors that influence the spatial distribution of the shrimp fleet. Prior studies have used geospatial regression models to investigate the regional (100s km^2^) and localized (10s km^2^) spatial dynamics of harvested populations, sometimes in relation to environmental factors [47, 48] but, to our knowledge, this is the first application of this approach to the spatial dynamics of a large-scale commercial fishery.

The spatial distribution of the shrimp fleet in relation to low bottom DO differed between the Louisiana shelf, a region that experiences recurrent, severe seasonal hypoxia, and the Texas shelf, a region that typically experiences limited hypoxia. The decline in shrimping effort at DO levels below 3 mg·L^-1^ in both regions is consistent with observations that the abundance of shrimp and other species is low in hypoxic waters due to avoidance behavior and shifts in spatial distributions [19, 20, 26, 27]. While shrimping effort declined sharply and monotonically at DO levels below 3 mg·L^-1^ on the Texas shelf, effort reached a minimum at 2 mg·L^-1^ and then increased slightly at lower DO levels on the Louisiana shelf. This difference in response to hypoxia between shrimpers on the Louisiana and Texas shelf is probably due to differences in the spatial extent of hypoxia in relation to the available shrimping grounds as well as to differences in the fishery between the two regions. The Louisiana shrimp fishery has historically been an estuarine and nearshore shelf fishery, with most of the shrimping effort occurring in nearshore waters that are susceptible to hypoxia [51]. Hypoxia in this region is driven by riverine nutrient inputs from the Mississippi-Atchafalaya River system and coastal plume dynamics that determine the amount and distribution of organic matter available to be respired (the hypoxic potential), as well as the area over which salinity stratification is sufficient to prevent ventilation of the bottom waters (the stratification envelope) [9]. The high sediment organic matter associated with this region fuels high benthic productivity that supports the foraging and growth of a number of demersal species [27, 39]. The high fishery production has led to this region being referred to as the “Fertile Fisheries Crescent.” [12]. Indeed, the general decline in shrimping effort with increasing DO on the Louisiana shelf (Fig 3E) is most likely because the fertile fisheries crescent and the hypoxic zone are spatially aligned at a course spatial scale. Because most regions of the shelf that support shrimp production are also susceptible to bottom water hypoxia, there may be limited alternative fishing locations for Louisiana shrimpers, particularly when hypoxia is severe. In contrast, the Texas fishery is managed via a seasonal closure that allows shrimp to emigrate to deeper, offshore waters and grow to larger sizes prior to harvest [51]. Hypoxia on the Texas shelf is more limited in spatial extent and typically occurs in the vicinity of major bays and from extension of the Mississippi-Atchafalaya River plume onto the northern Texas shelf [52, 53]. Less severe hypoxia and management that results in shrimp harvest in deeper offshore shelf waters may limit the effects of low DO on the spatial dynamics of shrimp and the shrimp fishery in this region.

It is unlikely that shrimpers are responding directly to low bottom DO but, rather, to hypoxia-induced shifts in the spatial distribution of their target species. Brown Shrimp can detect and avoid waters with low DO, and their estimated avoidance threshold based on field data in this region is 1.31 mg·L^-1^ [19, 20, 54]. Even though the Louisiana shelf is an open ecosystem, a number of hypoxia-avoiding species, including Brown Shrimp, aggregate at high density in close proximity (< 5 km) to hypoxic bottom waters. Hence, there are harvestable concentrations of shrimp in or near low DO waters on the Louisiana shelf that likely attract shrimpers to these areas. While the highest densities of Brown Shrimp nearly always occur within close proximity to the edge of the hypoxic zone, not all habitats near the edge harbor high shrimp densities, suggesting hypoxia may also induce small-scale patchiness in local shrimp abundance [19]. A similar effect of higher average catch but increased variation in catch has been shown for trawl fisheries operating near the edges of marine protected areas [55] and associated with frontal concentrations of fish along marginal ice zones [56]. Low abundance of shrimp in severely hypoxic water and increased patchiness near the edges of the hypoxic zone may require increased search time for shrimpers to locate productive fishing areas, perhaps accounting for the increase in shrimping effort in low DO waters off Louisiana.

In contrast to the Louisiana shelf, the monotonic shift in shrimping effort from inshore to offshore waters across the Texas shelf is consistent with avoidance of limited nearshore hypoxia. Shrimp landings on the Texas shelf are positively correlated with landings on the Louisiana shelf, and the strength of this relationship increases during periods of severe hypoxia, suggesting the population and fishery dynamics are linked between the two regions [57]. Zimmerman and Nance [57] postulated that extensive hypoxia along the Louisiana coast impedes the offshore migration of shrimp to deeper shelf waters, forcing alongshore migration to the northern Texas coast. While the average effect of hypoxia on the distribution of shrimping effort on the Texas shelf over the seven years of ELB data was a shift to offshore waters, during the year of most extensive hypoxia (2008), shrimping effort was highest close to shore. Perhaps high inshore shrimp densities due to alongshore emigration of shrimp originating from Louisiana waters resulted in the high inshore shrimping effort off Texas during 2008. Using oxygen isotopes DiMarco et al. [58] showed that in years of high Mississippi-Atchafalaya River flow, most of the fresh water on the northern Texas shelf originates from the westward extension of the Mississippi-Atchafalaya plume, while in years of low river flow fresh water was mostly from local riverine inputs. While currently not well-understood, annual differences in the sources of fresh water and nutrients driving hypoxia on the Texas shelf probably have consequences for the spatial configuration of hypoxia off Texas as well as emigration patterns of shrimp that, in turn, influence the spatial dynamics of the Texas fishery.

The direction and magnitude of hypoxia effects on shrimping effort on the Louisiana shelf also varied spatially but did not show consistent inshore-offshore shifts as in Texas waters. When hypoxia was severe, shrimping effort shifted to (1) offshore waters away from the hypoxic zone, (2) shallow, nearshore waters west of the Atchafalaya River mouth, and (3) a small area of typically oxygenated shelf water offshore of Terrebonne Bay. Prior studies have shown shifts in the distribution of Brown Shrimp to waters both inshore and offshore of the hypoxic zone [19, 20], consistent with the inshore and offshore shifts in the distribution of shrimping effort shown here. Further, a westward shift to shelf waters west of Atchafalaya Bay in response to severe hypoxia has been shown for flatfish [18] and for Gulf menhaden [29]. The shift in shrimping effort to a small region of the shelf south of Terrebonne Bay is particularly interesting because this area marks the approximate dividing line between two regions of hypoxia dominated by Mississippi River delta discharge to the east and Atchafalaya Bay discharge to the west [9]. This area of the shelf often has higher bottom DO levels than regions to the east or west and may function as an oxygenated refuge habitat for shrimp and other species in some years. Though data were limited, the increase in shrimping effort east of the Mississippi River delta and decrease to the west of the delta when hypoxia was severe, suggests the shrimp fleet may respond to hypoxia at this larger spatial scale as well. Collectively, our results indicate a complex response of the shrimp fleet to hypoxia on the Louisiana shelf that is comprised of both alongshore and cross shelf shifts in spatial distribution at multiple spatial scales.

The approach developed here to investigate hypoxia effects on the shrimp fishery was limited by several sources of uncertainty about the DO conditions that were experienced by shrimpers. The geographic location of individual shrimp tows was defined based on starting latitude and longitude, but tow durations (and hence tow distances) can be long and vary in direction [41]. Also, only a snapshot of DO from a concurrent hydrographic survey was available to characterize bottom DO conditions across the shelf. While our interpolation and censoring procedure attempted to align measures of shrimping effort and bottom water DO in space and time, higher-resolution environmental data at spatial and temporal scales synoptic with the fishery would help to better understand factors driving spatial patterns in shrimping effort. Similarly, the ability to link fine-scale effort data with actual shrimp catches would provide information on the mechanisms through which DO influences shrimping behavior.

The measure of shrimping effort (total tow hours) used in this study integrated the number of shrimp tows (tow density) and the duration that the nets were deployed (tow duration). Models that separated these two components of effort indicated that tow density rather than tow duration was the primary driver of the relationship between total shrimping effort and bottom water DO. The peak in tow density at moderately low DO (2.5–3 mg·L^-1^) suggests shrimpers aggregate in waters near the edge of the hypoxic zone where prior studies have indicated the highest densities of shrimp occur [27]. The more closely spaced effort contours in the severely hypoxic year (Fig 5) compared to the moderately hypoxic year (Fig 6) is consistent with hypoxia-induced aggregation of the shrimp fleet as well. Shrimpers typically make short exploratory tows with small try nets to find fishable densities of shrimp prior to deploying larger fishing nets [41]. The sharp drop in tow duration at the lowest DO levels on the Louisiana shelf is consistent with this searching behavior, perhaps due to imperfect knowledge about the spatial distribution of low DO, or in response to small-scale patchiness in shrimp densities in the vicinity of the hypoxic zone. Alternatively, shrimp densities and catch rates may be particularly high in this region leading to shorter tow durations due to gear saturation effects. Information on catch associated with tows in different DO conditions is needed to distinguish these possibilities. The peak in the number of shrimp tows and the slightly longer tow duration near the hypoxic zone suggests the potential for both competitive and cooperative effects among shrimp vessels, as has been shown in a number of other fisheries [59–62].

Currently, it is not clear whether the effects of low bottom DO on the spatial dynamics of the shrimp fleet are a net cost or a net benefit to the fishery. The fine-scale behavioral responses of the shrimp fleet to hypoxia on the Louisiana shelf suggest the possibility that shrimpers target hypoxia-induced aggregations of shrimp and may experience increased catchability [19], effects that would increase short-run profits to the fishery. Alternatively, shrimpers may experience increased search time or travel costs to locate areas with harvestable concentrations of shrimp, effects that would decrease short-run profits to the fishery. For example, in the North Carolina Brown Shrimp fishery, the integrated effects of hypoxia on shrimp growth, mortality, and catchability to the fishery lead to a net decrease in catches (13%) and profits (2.5%) [63, 64]. Depending on the relative strength of hypoxia effects on aspects of shrimp production and the behavioral response of shrimpers, overall profitability of the Gulf shrimp fishery could increase or decrease. Similarly, tradeoffs between the effects of riverine nutrient inputs and bottom water hypoxia on shrimp production may exist as well. Efforts to reduce nutrient inputs from the watershed in order to reduce the severity of hypoxia may have both positive and negative effects on the underlying basis of shrimp production, and should be considered in quantifying the economic effects of hypoxia and nutrient remediation strategies on the fishery.

Most work on fleet dynamics to date indicates that spatial patterns in fishing effort are related to catch, economic costs, and social factors [65–67]. The few studies that have addressed the effects of dynamic oceanographic factors on spatial patterns in fishing activity have mostly focused on pelagic fisheries (e.g., tuna) and environmental conditions in surface waters (e.g., temperature, chlorophyll) at ocean basin scales [68–71]. In these studies, the importance of environmental factors varies among fisheries and with the spatial scale of investigation, but in some cases are of similar importance to economic and social factors [71]. In economic models of fine-grained spatial fishing behavior, environmentally-driven effects are captured implicitly; recent past location-specific revenues and fishing location choices are predictors of current location choices [72–75]. Shrimpers in the northern Gulf of Mexico likely have general knowledge of the severity of hypoxia during summer based on spring river flow and precipitation conditions in the watershed, as well as its general location based on bathymetry, bottom topography, and experience from past fishing seasons. However, more specific knowledge in a given fishing season is probably based on recent experience (i.e., catch rates). It appears likely that hypoxia alters catchability to the fishery, at least at local scales, suggesting a decoupling of shrimp catch rates from actual abundance, which can lead to unintended management consequences [76–79]. As a result, further work is needed to determine the effects of hypoxia on the fishery and whether consideration in future management is warranted.
